# TNF-α augmented *Porphyromonas gingivalis* invasion in human gingival epithelial cells through Rab5 and ICAM-1

**DOI:** 10.1186/s12866-014-0229-z

**Published:** 2014-09-03

**Authors:** Yoshiko Kato, Makoto Hagiwara, Yuichi Ishihara, Ryutaro Isoda, Shinsuke Sugiura, Toshinori Komatsu, Naoyuki Ishida, Toshihide Noguchi, Kenji Matsushita

**Affiliations:** Department of Oral Disease Research, National Center of Geriatrics and Gerontology, Obu, Aichi 747-8511 Japan; Department of Periodontology, Aichigakuin University, Nagoya, Aichi Japan

**Keywords:** *Porphyromonas gingivalis*, Endocytosis, TNF-α, Rab5, ICAM-1, Persistent infection

## Abstract

**Background:**

Tumor necrosis factor alpha (TNF-α) plays a central role in the initiation and maintenance of immune responses to periodontopathic bacteria. However, excess TNF-α leads to dysregulated immune responses and progression of periodontitis. *Porphyromonas gingivalis* (*P. gingivalis*) invades gingival epithelial cells and then multiplies and survives for a long period. Additionally, increment of TNF-α in periodontal sites is associated with a high prevalence of gram-negative anaerobes such as *P. gingivalis*. However, it has not been determined whether TNF-α affects invasion of *P. gingivalis* in periodontal tissues.

**Results:**

We examined the effect of TNF-α on invasion of *P. gingivalis* in gingival epithelial cells and clarified the mechanism by which TNF-α augments invasion of *P. gingivalis.* Invasion of *P. gingivalis* into Ca9-22 cells was augmented by stimulation with TNF-α and it was inhibited by treatment with an antibody to TNF receptor-1. TNF-α increased production of ICAM-1, and *P. gingivalis* invasion was inhibited by an antibody to ICAM-1 in Ca9-22 cells. Silencing of Rab5 mRNA inhibited *P. gingivalis* invasion. Furthermore, the JNK inhibitor SP600125 inhibited invasion of *P. gingivalis* and also decreased the active form of Rab5 in Ca9-22 cells.

**Conclusion:**

TNF-α augments invasion of *P. gingivalis* in human gingival epithelial cells through increment of ICAM-1 and activation of Rab5. These phenomena may contribute to persistent infection of *P. ginigvalis* and prolongation of immune responses in periodontal tissues.

**Electronic supplementary material:**

The online version of this article (doi:10.1186/s12866-014-0229-z) contains supplementary material, which is available to authorized users.

## Background

Chronic periodontitis is initiated by a bacterial biofilm commonly called dental plaque, which initiates inflammation that affects the supporting structures of teeth, leading to bone and eventually tooth loss. The development of periodontitis is a multifactorial process involving interactions between the host and microorganisms that colonize the gingival sulcus. *Porphyromonas gingivalis* is a gram-negative anaerobe of dental plaque and it has been strongly implicated in the initiation and progression of periodontal disease and possesses a sophisticated array of virulence factors, including those that allow the bacterium to adhere to and invade host epithelial cells [1–5]. *P. gingivalis* invasion is accomplished by manipulating host signal transduction and remodeling of the cytoskeletal architecture. However, the molecular mechanisms used by *P. gingivalis* to facilitate internalization are only partially understood.

Intracellular bacterial pathogens have evolved highly specialized mechanisms to enter and survive intracellularly within their eukaryotic hosts. Rabs play an essential role in both endocytic and exocytic traffic in eukaryotic cells [6]. Rab5, one of the most studied Rab proteins in recent years, is involved in early steps of the endocytic process. Rab5 regulates intracellular membrane trafficking of several pathogens, including *Salmonella enterica* serovar Typhimurium [7–9], *Mycobacterium* spp [10], and *Listeria monocytogenes* [11]. Rab5 may also mediate internalization of *P. gingivalis* in host cells; however, little is known about the role of Rab5 in *P. gingivalis* invasion.

TNF-α is a potent pleiotropic proinflammatory cytokine and is released by a variety of different cell types in response to various stimuli, including bacteria, parasites, viruses, cytokines and mitogens. TNF-α is involved in systemic and local inflammation due to stimulation of different signal transduction pathways, inducing the expression of a broad range of genes. TNF-α regulates a host response to infection; on the other hand, inappropriate expression of TNF-α has detrimental effects for the host. Deregulation of TNF-α has been implicated in the pathogenesis of numerous complex diseases, including periodontitis [12–14], cardiovascular diseases [15,16], diabetes mellitus [17,18], autoimmune diseases [19,20], and cancer [21,22]. Clinical studies have shown an upregulation of TNF-α in periodontitis, e.g., in gingival crevicular fluid [23], in gingival tissues [24], and in plasma and serum [14,25]. TNF-α was shown to have an impact on different biological processes, including induction of inflammatory mediators, such as matrix metalloproteases (MMPs), cytokines, chemokines and prostaglandins [26], endothelial cell activation and endothelial-leukocyte interactions [27], monocyte adhesion [28], mediating bone remodeling [29], and oxidative processes [30]. *P. gingivalis* induces highest levels of TNF-α expression, followed by IL-1 and IL-6 [31]. However, we have no information on whether TNF-α affects invasion of *P. gingivalis* in periodontal tissues. In the present study, we examined the effect of TNF-α on invasion of *P. gingivalis* in gingival epithelial cells and clarified the molecular mechanism by which TNF-α augments invasion of *P. gingivalis.*

## Results

### TNF-α augments invasion of *P. gingivalis* in gingival epithelial cells

**Figure 1:**
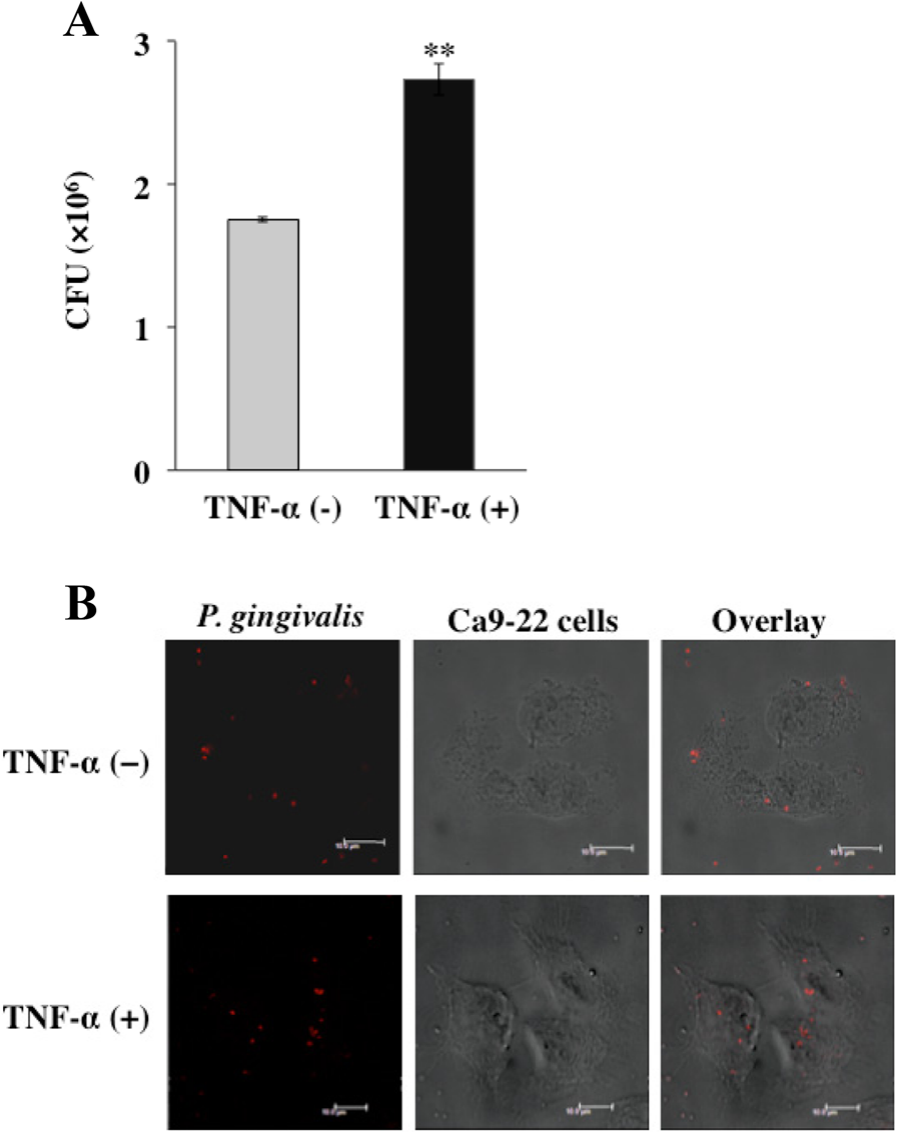
**TNF-α augments invasion of** ***P. gingivalis*** **in Ca9-22 cells. (A)** Ca9-22 cells were treated with 10 ng/ml of TNF-α for 3 h. The cells were further incubated with *P. gingivalis* ATCC 33277 at an MOI of 100 for 1 h. Media in the cultures were then replaced with new media containing antibiotics for 1 h. Lysates of the cells with sterile water were then seeded on horse blood agar plates to determine the numbers of viable intracellular bacteria (means ± standard deviations [SD] [n = 3]). **, *P* < 0.01 versus TNF-α (−). CFU: colony forming units. **(B)** Ca9-22 cells were treated with 10 ng/ml of TNF-α for 3 h and were then incubated with *P. gingivalis* ATCC 33277 for 1 h. *P.gingivalis* was stained using antiserum for *P. gingivalis* whole cells. Then localization of *P. gingivalis* in the cells was observed by a confocal laser scanning microscope. Each molecule was visualized as follows: *P. gingivalis* (red). Bars in each panel are 10 μm.

We first examined the effect of TNF-α on invasion of *P. gingivalis* in Ca9-22 cells. The cells were treated with 10 ng/ml of TNF-α for 3 h and were then incubated with *P. gingivalis* (MOI =100) for 1 h. Invasion of the cells by *P. gingivalis* was determined by an invasion assay. Invasion of Ca9-22 cells by *P. gingivalis* was observed without TNF-α pretreatment. However, the invasion was significantly increased by stimulation with TNF-α (Figure 1A). We also observed localization of intracellular *P. gingivalis* in the cells by using a confocal laser scanning microscope. Z-stack image of the cells shows the intracellular localization of *P. gingivalis*. Intracellular *P. gingivalis* was increased by stimulation with TNF-α, although a small amount of *P. gingivalis* was found without TNF-α pretreatment (Figure 1B).

### TNF-α-augmented invasion of *P. gingivalis* is mediated by TNF receptor-I

**Figure 2:**
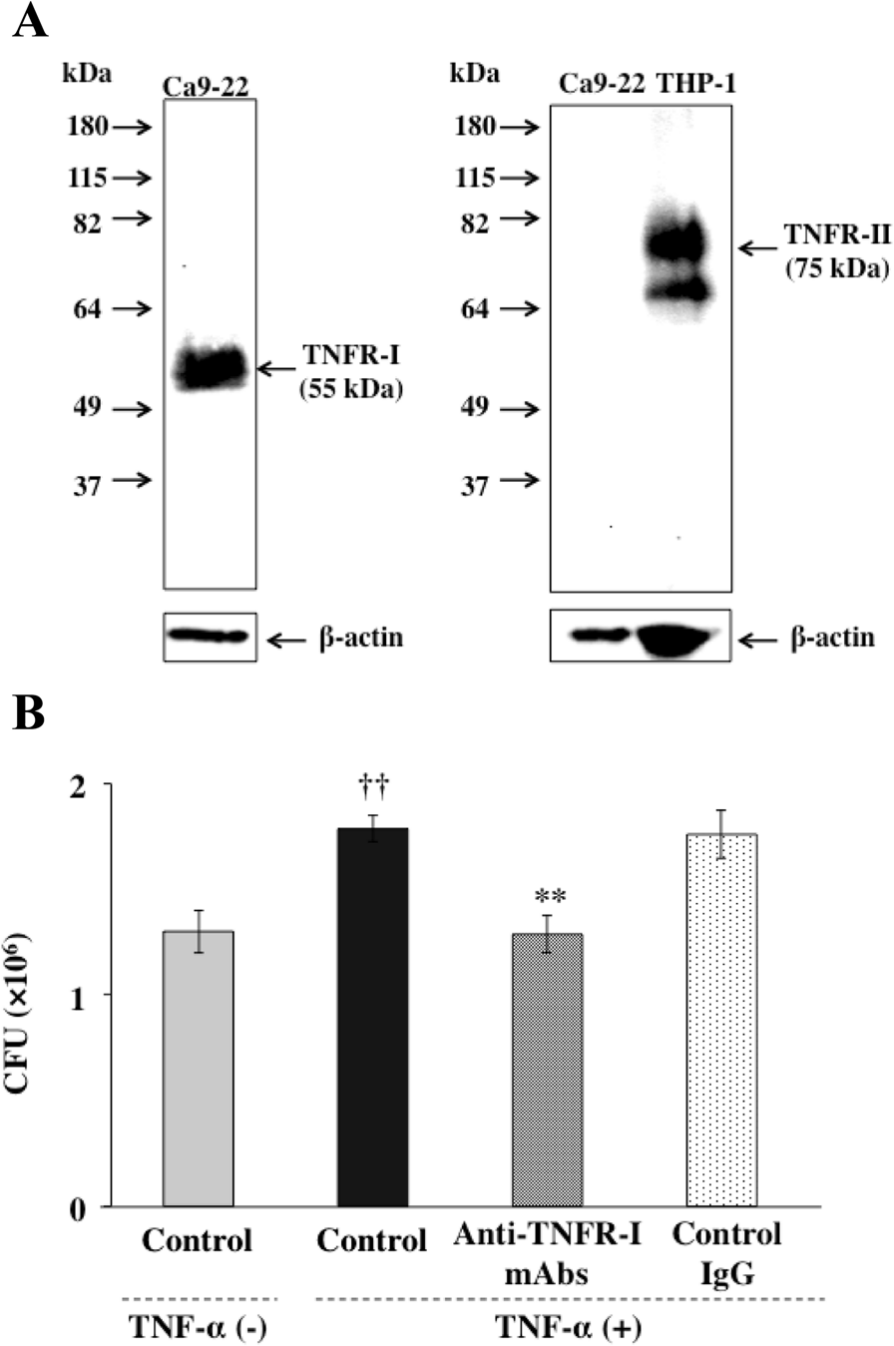
**TNF-α-augmented invasion of** ***P. gingivalis*** **is mediated by TNF receptor-I. (A)** Expression of TNF receptors on Ca9-22 cells. Expression of TNF receptors in lysates of the cells was analyzed by Western blotting with anti-TNFR-I and anti-TNFR-II monoclonal antibodies. Human monocytic THP-1 cells were used as a positive control of TNFR-II. **(B)** Anti-TNFR-I antibody blocked TNF-a-augmented invasion of *P. gingivalis* in Ca9-22 cells. Ca9-22 cells were preincubated with 5 μg/ml of anti-TNFR-I monoclonal antibody or mouse IgG at 37°C for 1 h and were then incubated with TNF-α for 3 h. The cells were further incubated with *P. gingivalis* (MOI =100) for 1 h. Viable *P. gingivalis* in the cells was determined as described in Methods. (Means ± standard deviations [SD] [n = 3]). ††, *P* < 0.01 versus control + TNF-α (−); **, *P* < 0.01 versus none + TNF-α (+).

The biological effects of TNF-α are transmitted via two distinct membrane receptors, TNFR-I and TNFR-II [32,33]. To determine which type of TNFR mediates *P. gingivalis* invasion in Ca9-22 cells, we examined the effects of neutralization of TNFRs on the TNF-α-augmented invasion of *P. gingivalis*. We first examined the expression of TNFR-I and TNFR-II in Ca9-22 cells by Western blotting. The cells expressed TNFR-I but not TNFR-II (Figure 2A). We next examined the effects of a neutralizing anti-TNFR-I mAb on the TNF-α-induced invasion of *P. gingivalis* in Ca9-22 cells. The cells were preincubated with a mouse monoclonal antibody to TNFR-I for 1 h. Then the cells were treated with TNF-α prior to addition of *P. gingivalis*. The anti-TNFR-I antibody exhibited a significant inhibitory effect on the invasion of *P. gingivalis* in Ca9-22 cells (Figure 2B). In contrast, a control mouse IgG antibody did not prevent the augmentation of *P. gingival*is invasion by TNF-α.

### TNF-α augments invasion of *P. gingivalis* through NF-κB and MAPK pathways

**Figure 3:**
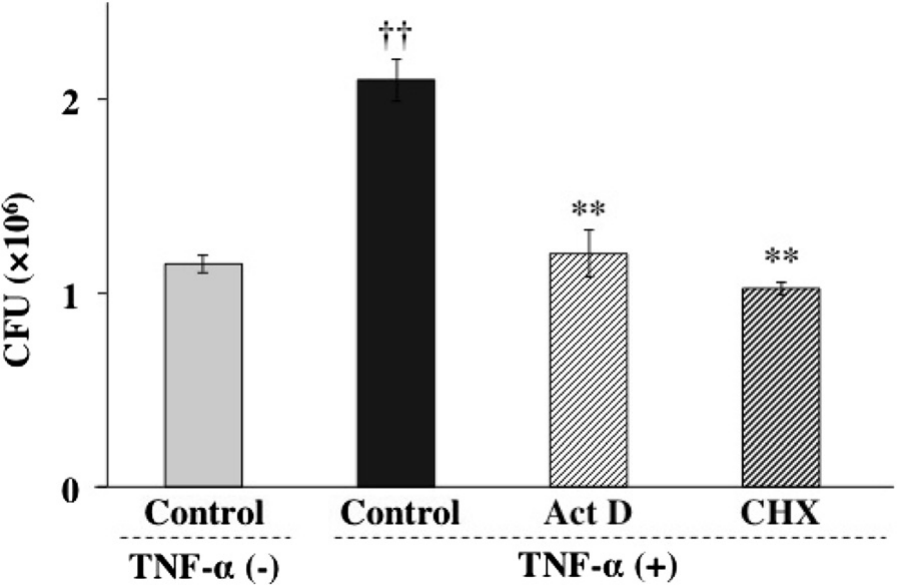
**TNF-α augments invasion of** ***P. gingivalis*** **through synthesis of mRNAs and proteins.** Actinomycin D and cycloheximide inhibited TNF-α-augmented invasion of *P. gingivalis* in Ca9-22 cells. Confluent Ca9-22 cells were preincubated with 1 μg/ml actinomycin D (Act D) or cycloheximide (CHX) at 37°C for 1 h and were then incubated with TNF-α for 3 h. The cells were further incubated with *P. gingivalis* (MOI =100) for 1 h. Viable *P. gingivalis* in the cells was determined as described in Methods. (Means ± standard deviations [SD] [n = 3]). ††, *P* < 0.01 versus control + TNF-α (−); **, *P* < 0.01 versus control + TNF-α (+).

**Figure 4:**
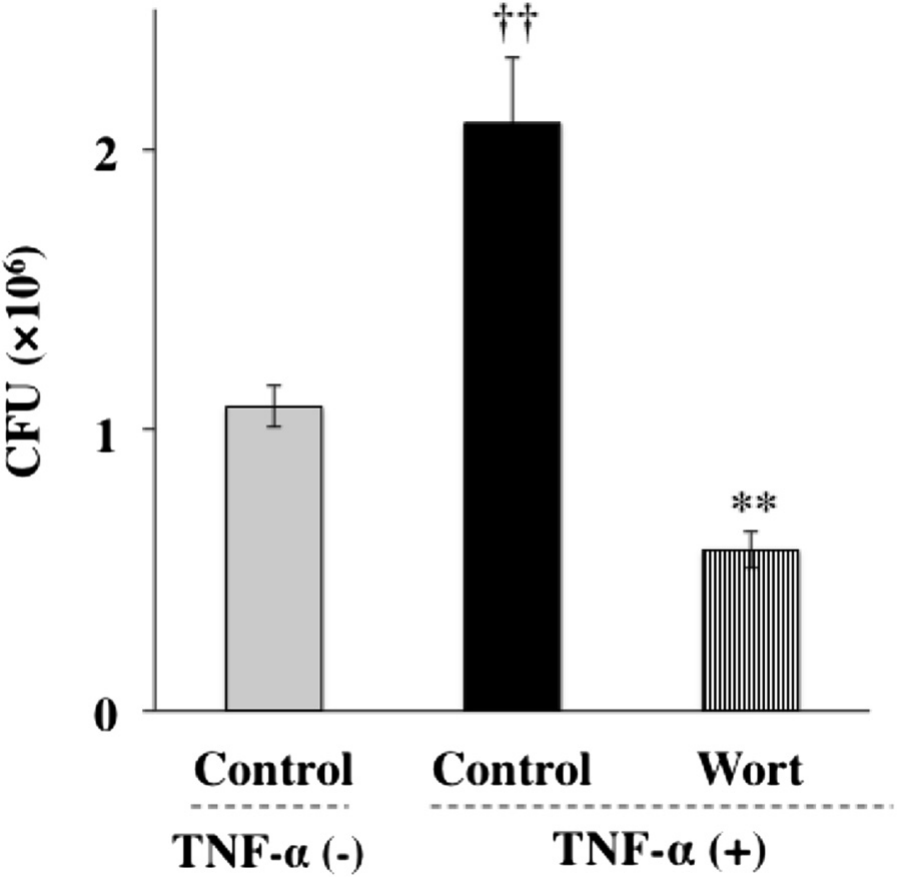
**TNF-α augments endocytosis of** ***P. gingivalis*** **through PI3K pathways.** A PI3K inhibitor suppressed TNF-a-augmented invasion of *P. gingivalis* in Ca9-22 cells. Ca9-22 cells were preincubated with wortmannin (Wort, 300 nM) at 37°C for 3 h and were then incubated with TNF-α. Viable *P. gingivalis* in the cells was determined as described in Methods. (Means ± standard deviations [SD] [n = 3]). ††, *P* < 0.01 versus control + TNF-α (−); **, *P* < 0.01 versus control + TNF-α (+).

**Figure 5:**
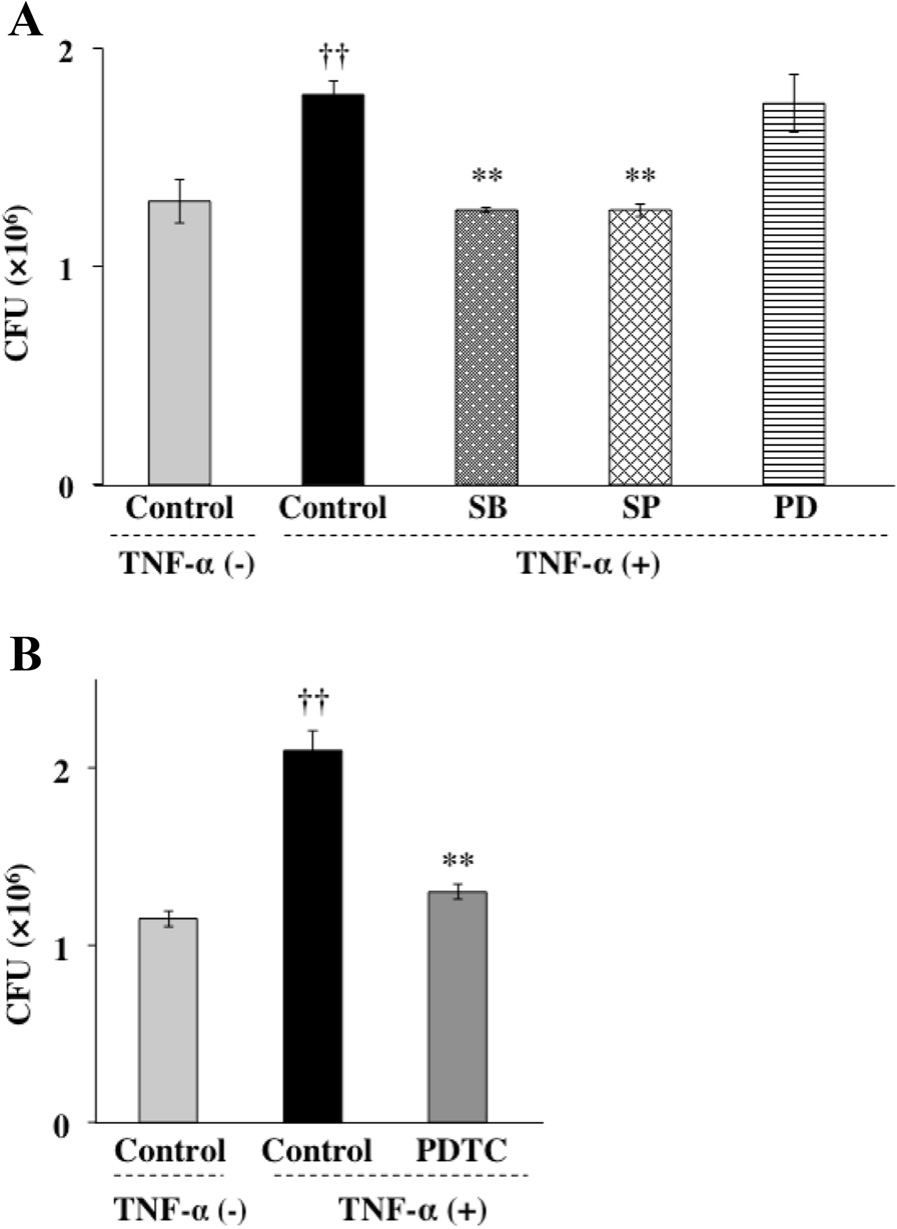
**TNF-α augments invasion of** ***P. gingivalis*** **through NF-kB and MAPK pathways. (A)** JNK and p38 inhibitors blocked TNF-a-augmented invasion of *P. gingivalis* in Ca9-22 cells. Confluent Ca9-22 cells were preincubated with MAP kinase inhibitors (p38 inhibitor (SB203580, 5 μM), JNK inhibitor (SP600125, 1 μM ) and ERK inhibitor (PD98059, 5 μM)) at 37°C for 1 h and were then incubated with TNF-α. Viable *P. gingivalis* in the cells was determined as described in Methods. (Means ± standard deviations [SD] [n = 3]). ††, *P* < 0.01 versus control + TNF-α (−); **, *P* < 0.01 versus control + TNF-α (+). **(B)** NF-κB inhibitor suppressed TNF-α-augmented invasion of *P. gingivalis* in Ca9-22 cells. Ca9-22 cells were preincubated with an NF-κB inhibitor (PDTC, 5 μM) at 37°C for 1 h and were then incubated with TNF-α. Viable *P. gingivalis* in the cells was determined as described in Methods. (Means ± standard deviations [SD] [n = 3]). ††, *P* < 0.01 versus control + TNF-α (−); **, *P* < 0.01 versus control + TNF-α (+).

To determine whether mRNA synthesis and protein synthesis were required for *P. gingivalis* invasion, Ca9-22 cells were preincubated with 1 μg/ml of the RNA polymerase II inhibitor actinomycin D or the protein synthesis inhibitor cycloheximide for 1 h and were then incubated with TNF-α prior to addition of *P. gingivalis*. Actinomycin D and cycloheximide exhibited significant inhibitory effects on the invasion of *P.gingivalis* into Ca9-22 cells (Figure 3). The PI3K/Akt signaling pathway is commonly initiated by transmembrane receptor signaling and controls cellular phagocytic responses through multiple downstream targets that regulate actin polymerization and cytoskeletal arrangements at the target site [34]. In addition, TNF-α activates the PI3K/AKT signaling pathway [35]. Therefore, we examined the relationship between PI3K activity and *P. gingivalis* invasion in Ca9-22cells. Ca9-22 cells were preincubated with wortmannin at 37°C for 3 h and were then incubated with TNF-α. Treatment with wortmannin also exhibited significant inhibitory activity towards the invasion of *P. gingivalis* enhanced by TNF-α (Figure 4). Several lines of evidence indicate that cellular effects of TNF-α were elicited through the activation of MAPK and NF-κB pathways. To explore the contribution of MAPK and NF-κB to TNF-α-augmented invasion of *P. gingivalis*, we examined whether *P. gingivalis* is able to invade Ca9-22 cells in the presence or absence of MAPK inhibitors and an NF-κB inhibitor. Ca9-22 cells were preincubated with a p38 inhibitor (SB 203580, 5 μM), JNK inhibitor (SP 600125, 1 μM), ERK inhibitor (PD 98059, 5 μM) or NF-κB inhibitor (PDTC, 5 μM) for 1 h and were then incubated with TNF-α prior to addition of *P. gingivalis*. SB 203580 and SP 600125 exhibited significant inhibitory effects on the invasion of *P. gingivalis* into Ca9-22 cells (Figure 5A). In contrast, PD 98059 did not prevent the invasion of *P. gingivalis* augmented by TNF-α. PDTC also exhibited significant inhibitory activity towards the invasion of *P. gingivalis* enhanced by TNF-α (Figure 5B). These results suggest that TNF-α augmented invasion of *P. gingivalis* is mediated by p38 and JNK pathways and activation of NF-κB.

### ICAM-1 mediates invasion of *P. gingivalis*

**Figure 6:**
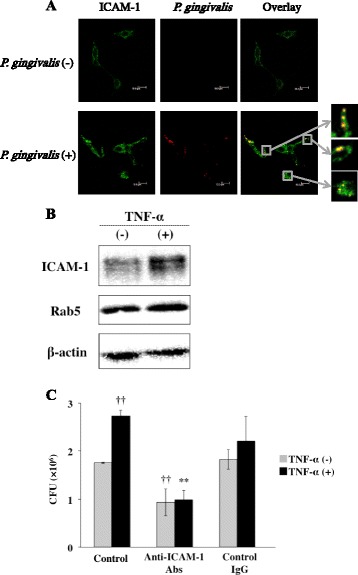
**ICAM-1 mediates invasion of** ***P. gingivalis.*** **(A)** Ca9-22 cells were incubated with *P. gingivalis* for 1 h. The cells were then stained using anti-ICAM-1 antibody. ICAM-1 is shown in green and *P. gingivalis* is shown in red. Bars in each panel are 10 μm. **(B)** TNF-α increased expression of ICAM-1 in Ca9-22 cells. Ca9-22 cells were treated with 10 ng/ml of TNF-α for 3 h. The cells were lysed and the expression of ICAM-1 and Rab5 was analyzed by Western blotting with antibodies for each molecule. **(C)** Antibody to ICAM-1 inhibits invasion of *P. gingivalis* in cells. Ca9-22 cells were treated with TNF-α for 3 h and were then incubated with an anti-ICAM-1 antibody or a control IgG antibody for 2 h. Viable *P. gingivalis* in the cells was determined as described in Methods. (Means ± standard deviations [SD] [n = 3]). ††, *P* < 0.01 versus control + TNF-α (−); **, *P* < 0.01 versus none + TNF-α (+).

Expression of ICAM-1 is required for invasion of some bacteria in KB cells [36]. To determine whether ICAM-1 affects *P. ginigvalis* invasion into cells, we first examined co-localization of *P. gingivalis* with ICAM-1 in cells. Ca9-22 cells were incubated with *P. gingivalis*, and localization of ICAM-1 and *P. ginigvalis* in the cells was observed by a confocal laser scanning microscope. ICAM-1 strongly expressed around the cell surface was partially co-localized with *P. gingivalis* in the cells (Figure 6A). We also examined the expression of ICAM-1 in TNF-α-treated Ca9-22 cells. Ca9-22 cells were treated with or without TNF-α for 3 h. The cells were lysed and expression of ICAM-1 was analyzed by Western blotting. ICAM-1 was expressed in Ca9-22 cells without TNF-α stimulation (Figure 6B). However, TNF-α increased the expression of ICAM-1 in the cells. We next examined whether ICAM-1 is associated with invasion of *P. gingivalis* into the cells. Ca9-22 cells were treated with TNF-α for 3 h, incubated with an anti-ICAM-1 antibody or a control IgG antibody for an additional 2 h, and then incubated with *P. gingivalis*. Anti-ICAM-1 antibody suppressed invasion of *P. gingivalis* in the cells with or without TNF-α pretreatment (Figure 6C). In contrast, *P. gingivalis* invasion was not prevented by control IgG. These results suggest that ICAM-1 is partially associated with invasion of *P. gingivalis* into Ca9-22 cells.

### Rab5 mediates endocytosis of *P. gingivalis*

**Figure 7:**
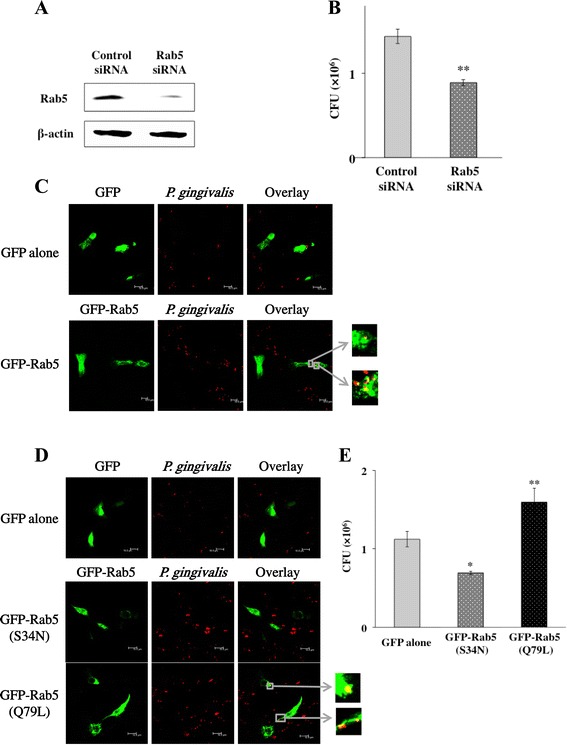
**Rab5 mediates endocytosis of** ***P. gingivalis.*** **(A)** Ca9-22 cells were transfected with 100 pmol siRNA specific for Rab5 or control siRNA using Lipofectamine 2000 reagent, as described by the manufacturer. Then expression of Rab5 in the cells was examined by Western blotting. **(B)** Rab5 siRNA-transfected Ca9-22 cells were incubated with *P. gingivalis* for 1 h. Viable *P. gingivalis* in the cells was determined as described in Methods. (Means ± standard deviations [SD] [n = 3]. **, *P* < 0.01 versus control siRNA. **(C)** Ca9-22 cells were transfected with expression vectors with inserted genes of GFP alone and GFP-Rab5. The cells were incubated with *P. gingivalis* for 1 h. The cells were then stained using anti-*P. gingivalis* antiserum. Each molecule was visualized as follows: GFP and GFP-Rab5 (green) and *P. gingivalis* (red). Bars in each panel are 10 μm. **(D)** Active form of Rab5 colocalizes with *P. gingivalis* in Ca9-22 cells. Ca9-22 cells were transfected with vectors with inserted genes of GFP alone (control), GFP-Rab5 (S34N) (inactive form of Rab5), and GFP-Rab5 (Q79L) (active form of Rab5). The cells were incubated with *P. gingivalis* for 1 h. Then localization of *P. gingivalis* and Rab5 in the cells was observed by a confocal laser scanning microscope. Each molecule was visualized as follows: GFP and GFP-Rab5 (green) and *P. gingivalis* (red). Bars in each panel are 10 μm. **(E)** Overexpression of the active form of Rab5 increased invasion of *P. gingivalis* in Ca9-22 cells. Ca9-22 cells were transfected with expression vectors with inserted genes of GFP alone (Control), GFP-Rab5 (S34N) and GFP-Rab5 (Q79L). Viable *P. gingivalis* in the cells was determined as described in Methods. (Means ± standard deviations [SD] [n = 3]). *, *P* < 0.05 versus control; **, *P* < 0.01 versus GFP alone.

Several studies have shown that Rab5 regulates events in the fusion of bacteria-containing vacuoles and early endosomes [37–39]. Therefore, we investigated whether Rab5 mediates *P. gingivalis* invasion into cells. We first examined the expression of Rab5 in Ca9-22 cells by Western blotting. As shown in Figure 6B, Rab5 was expressed in Ca9-22 cells. However, the level of expression was not affected by TNF-α. We next investigated the role of Rab5 in *P. gingivalis* invasion using an siRNA interference approach. Invasion assays were carried out following transfection of Rab5-specific siRNA at a concentration of 100 pmol for 24 h. Then expression of Rab5 in the cells was examined by Western blotting (Figure 7A). The Rab5 siRNA-transfected Ca9-22 cells were incubated with *P. gingivalis* for 1 h. Internalization of *P. gingivalis* into the cells was reduced by silencing the Rab5 gene (Figure 7B). To determine whether the Rab5 affects *P. ginigvalis* invasion into cells, Ca9-22 cells expressing GFP-Rab5 were treated with *P. gingivalis*, and localization of Rab5 and *P. ginigvalis* in the cells was observed by a confocal laser scanning microscope. Transfected GFP-Rab5 was partially co-localized with *P. gingivalis* in the cells (Figure 7C). These results suggest that Rab5 is partially associated with invasion of *P. gingivalis* into Ca9-22 cells.

### Overexpression of the active form of Rab5 increased invasion of *P. gingivalis*

Rab5 proteins switch between two distinct conformations, an active state characterized by binding to GTP and an inactive state bound to GDP. To test whether the activity of Rab5 affects *P. ginigvalis* invasion into cells, Ca9-22 cells expressing fluorescent-labeled GFP alone (control), GFP-Rab5 (S34N) (constitutively inactive mutant), and GFP-Rab5 (Q79L) (constitutively active mutant) were treated with *P. gingivalis*, and localization of Rab5 and *P. ginigvalis* in the cells was observed by a confocal laser scanning microscope. Transfected GFP-Rab5 (Q79L) was co-localize with *P. gingivalis* in the cells (Figure 7D). In contrast, GFP-Rab5 (S34N) did not co-localize with *P. gingivalis* in the cells. We next transfected vectors expressing GFP alone, GFP-Rab5 (S34N) and GFP-Rab5 (Q79L) into Ca9-22 cells. The transfected cells were then treated with *P. ginigvalis* and the levels of invasion were compared among those cells. Internalization of *P. gingivalis* into cells was increased in Ca9-22 cells expressing GFP-Rab5 (Q79L) compared to that in Ca9-22 cells expressing GFP alone (Figure 7E). On the other hand, overexpression of GFP-Rab5 (S34N) suppressed invasion of *P. gingivalis* into the cells. These results suggest that the activity of Rab5 influences *P. gingivalis* invasion.

### TNF-α was associated with activity of Rab5 through the JNK pathway

**Figure 8:**
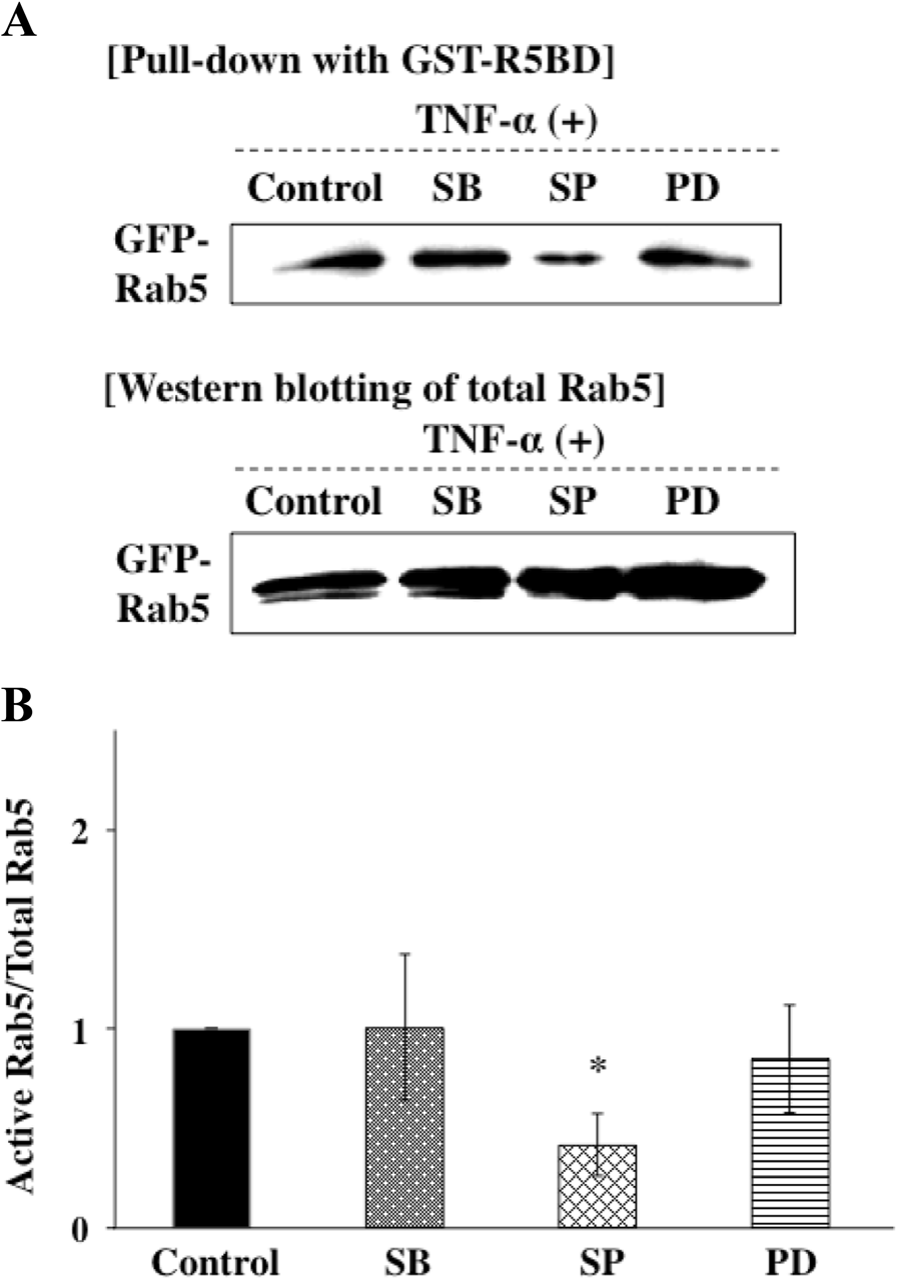
**TNF-α was associated with activity of Rab5 through the JNK pathway. (A)** Ca9-22 cells were transfected with an expression vector with inserted GFP-Rab5 gene. The transfected cells were preincubated with MAP kinase inhibitors, including a p38 inhibitor (SB203580, 5 μM) (indicated as “SB”), JNK inhibitor (SP600125, 1 μM) (indicated as “SP”) and ERK inhibitor (PD98059, 5 μM) (indicated as “PD”), at 37°C for 1 h and were then incubated with TNF-α for 3 h. The active form of Rab5 in the cell lysates was subjected to a GST-R5BD pull-down assay and was analyzed by Western blotting with anti-GFP antibodies as described in Methods. **(B)** Level of the active form of Rab5-GTP was normalized to total GFP-Rab5 and quantified by a densitometer. (Means ± deviations [SD] [n = 3]). *, *P* < 0.05 versus control.

**Figure 9:**
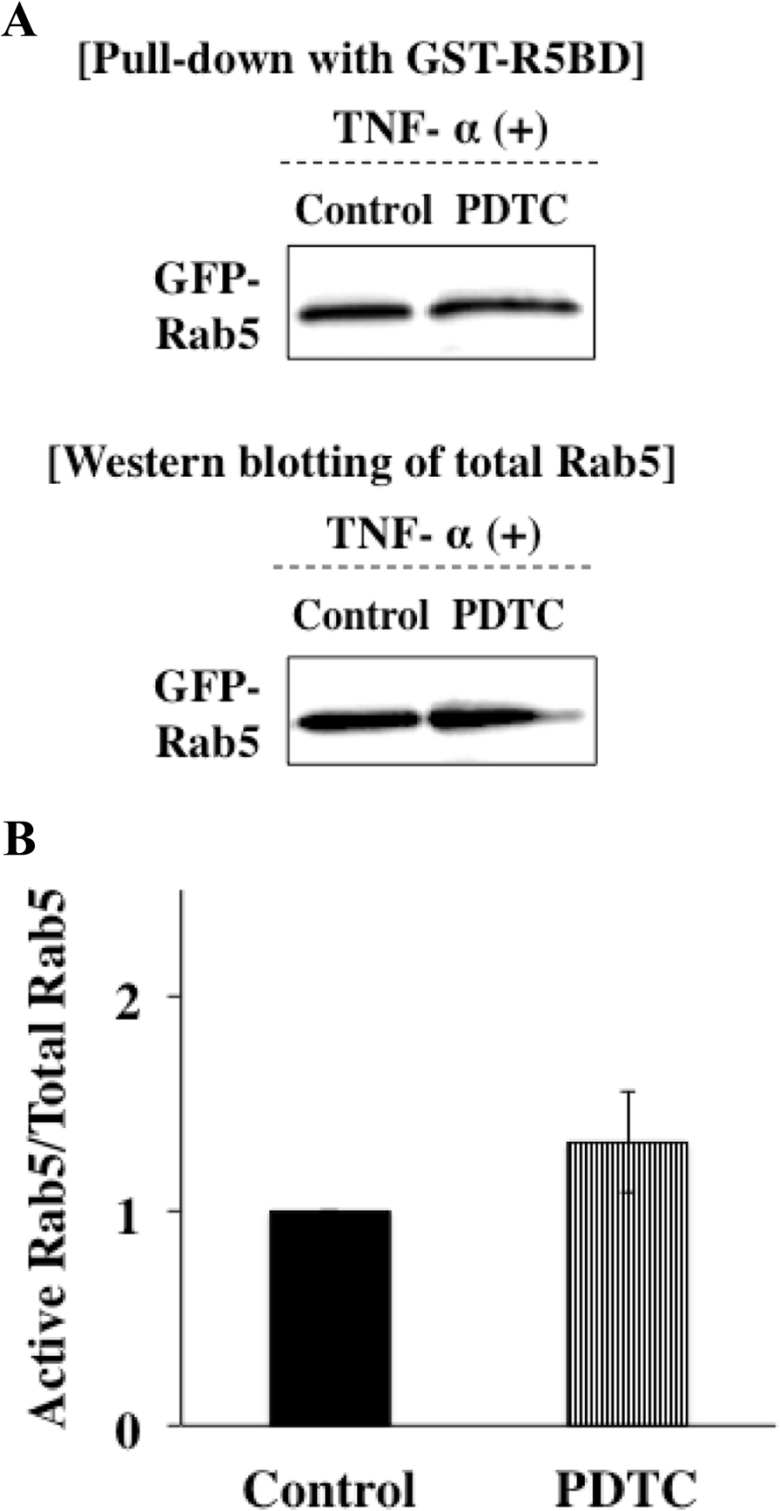
**TNF-α was not associated with activity of Rab5 through the NF-κB pathway. (A)** Ca9-22 cells were transfected with an expression vector with an inserted GFP-Rab5 gene. The transfected cells were preincubated with an NF-κB inhibitor (PDTC, 5 μM) at 37°C for 1 h and were then incubated with TNF-α for 3 h. The active form of Rab5 in the cell lysates was subjected to a GST-R5BD pull-down assay and was analyzed by Western blotting with anti-GFP antibodies as described in Methods. **(B)** Level of the active form of Rab5-GTP was normalized to total GFP-Rab5 and quantified by a densitometer. (means ± deviations [SD] [n = 3]).

Several cytokines can control the activity of Rab5 to regulate the rate of endocytosis through activating the downstream signaling pathway. Therefore, we examined whether activation of Rab5 was affected by MAP kinases activated with TNF-α signals using a pull-down approach with a fusion protein that selectively binds GTP-loaded Rab5 (GST-R5BD). The system selectively bound GTP-bound Rab5 (active form of Rab5). Ca9-22 cells were transfected with an expression vector with inserted GFP-Rab5 gene. The transfected cells were preincubated with MAP kinase inhibitors, including a p38 inhibitor (SB203580), JNK inhibitor (SP600125) and ERK inhibitor (PD98059), and were then incubated with TNF-α. The active form of Rab5 in the cell lysates was subjected by a GST-R5BD pull-down assay and was analyzed by Western blotting. Level of the active form of Rab5 induced by TNF-α was not affected by treatments with SB203580 and PD98059. However, treatment with SP60015 decreased the level of the active form of Rab5 induced by TNF- (Figure 8A, B). These results suggest that JNK kinase mediates activation of Rab5 by stimulation with TNF-α. Furthermore, we invastigated whether NF-kB inhibition affects the activation of Rab5. Ca9-22 cells were transfected with an expression vector with an inserted GFP-Rab5 gene. The transfected cells were preincubated with an NF-κB inhibitor (PDTC, 5 μM) at 37°C for 1 h and were then incubated with TNF-α for 3 h. The active form of Rab5 in the cell lysates was subjected to a GST-R5BD pull-down assay and was analyzed by Western blotting with anti-GFP antibodies. Treatment with PDTC also did not affect the level of the active form of Rab5 induced by TNF- (Figure 9A, B). These results suggest that NF-κB does not mediate activation of Rab5 by stimulation with TNF-α.

### TNF-α increased colocalization of *P. gingivalis* with ICAM-1 and Rab5

**Figure 10:**
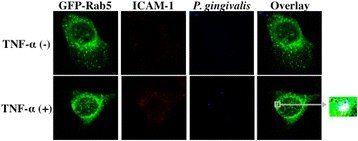
**TNF-α increased colocalization of** ***P. gingivalis*** **with ICAM-1 and Rab5.** Ca9-22 cells were transfected with expression vectors with inserted genes of GFP-Rab5. The cells were treated with TNF-α for 3 h and were further incubated with *P. gingivalis* for 1 h. The cells were then stained using an anti-ICAM-1 antibody and anti-*P. gingivalis* antisera. Each molecule was visualized as follows: GFP-Rab5 (green), ICAM-1 (red), and *P. gingivalis* (blue).

Finally, we examined the relationships among *P. gingivalis*, ICAM-1 and Rab5 in Ca9-22 cells. Ca9-22 cells were transfected with expression vectors with inserted genes of GFP-Rab5 and were then treated with TNF-α and further incubated with *P. gingivalis*. The cells were then stained using an anti-ICAM-1 antibody and antiserum to *P. gingivalis* whole cells. A small amount of *P. gingivalis* that co-localized with ICAM-1 and GFP-Rab5 was observed in Ca9-22 cells without TNF-α stimulation. However, TNF-α stimulation increased co-localization of *P. gingivalis*, ICAM-1 and GFP-Rab5 in Ca9-22 cells (Figure 10). These findings suggest that TNF-α affects the localization of Rab5 and ICAM-1 in cells and may enhance internalization of *P. gigivalis* in the cells.

## Discussion

TNF-α is a potent pleiotropic proinflammatory cytokine and has been implicated in the pathogenesis of periodontitis [12–14]. TNF-α was also shown to activate oral epithelial cells. However, it was not known whether TNF-α affects *P. gingivalis* invasion in epithelial cells. In the present study, we demonstrated for the first time that TNF-α augmented *P. gingivalis* invasion in oral epithelial cells.

In this study, we showed that TNF-α activated Rab5 through JNK but not through p38 and ERK, although TNF-α activates all of them. Activation of JNK is associated with the invasive process of *P. gingivalis* [1,40]. Therefore, JNK activated by TNF-α may mediate activation of Rab5 and may enhance internalization of *P. gingivalis* in cells. Rab5 is an important regulator of early endosome fusion. Therefore, TNF-α may induce formation of early phagosomes by activating Rab5. On the other hand, Bhattacharya et al. [41] demonstrated that cytokines regulate bacterial phagocytosis through induction of Rab GTPases. They showed that IL-6 specifically induces the expression of Rab5 and activates Salmonella trafficking in cells through ERK activation. On the other hand, IL-12 induced Rab7 expression through p38. Another study showed that activation of p38 MAPK regulates endocytosis by regulating the activity of Rab5 accessory proteins such as Rab5-GDI, EEA1, and rabenosyn-5, which are known to regulate membrane transport during endocytosis. Several independent studies have also shown that activation of ERK regulates endocytic traffic of multiple receptor systems, for example, 5-HT_1A_ receptor, m1 muscarinic receptor, and opioid receptors [42–45]. These findings suggest that activation of different kinases regulates intracellular trafficking and also indicate that the mechanism by which MAPKs regulate endocytosis may differ depending on the stimulants and cells.

As shown in Figure 5B, the p38 inhibitor SB203580 blocked TNF-α-augmented *P. gingivalis* invasion in Ca9-22 cells. However, SB203580 did not inhibit the activation of Rab5 despite the fact that internalization of *P. gingivalis* into the cells was partially blocked by knock-down of Rab5a. TNF-α induced ICAM-1 expression through activating ERK/p38 MAPK [46]. Therefore, p38 inhibition suppressed ICAM-1 expression followed by decrease in *P. gingivalis* invasion. On the other hand, Rab5 has three isoforms (A, B, and C) and the isoforms are able to compensate for each other. As we interfered with the expression of Rab5a but not that of Rab5b and 5c, Rab5b and Rab5c, which were not blocked, may compensate the function of Rab5a for bacterial internalization.

*P. gingivalis* can enter Ca9-22 cells without TNF-α stimulation (Figure 1A). Blockade of the TNF receptor and inhibition of p38 and JNK did not completely inhibit *P. gingivalis* invasion. These results suggest that *P. gingivalis* is also internalized in a TNF-α-independent manner. *P. gingivalis* invades gingival epithelial cells without any stimulation to the host cells. *P. gingivalis* fimbriae interact with cell surface molecules such as integrins and the interactions trigger colonization and internalization of the bacteria in various cells [47,48]. Furthermore, the trypsin-like cysteine protease gingipain produced by *P. gingivalis* also plays an important role during *P. gingivalis* entry into cells [47]. *P. gingivalis* can enter host cells by using these molecules without TNF-α stimulation. However, TNF-α is increased in inflamed periodontal tissues and gingival crevicular fluids. In those tissues, *P. gingivalis* invasion is increased, and it promotes persistent infection and avoids immune surveillance. The cellular tropism of *P. gingivalis* depends in part upon the fimbriase of the bacteria and the receptors of the host cell. We used Ca9-22 cells as a model for gingival cell infection. These cells were originally derived from human gingival carcinoma and phenotypically resemble gingival epithelial cells. However, Ca9-22 cells may also express some cell surface receptors that are different from endogenous gingival cells. Thus our experimental system is representative of bacteria-host interactions in vivo, but not a perfect model We have little evidence about that in vivo and further study is needed to make a final conclusion concerning the physiological relevance of the phenomena.

Ca9-22 cells expressed TNFR-I but not TNFR-II (Figure 2A). We also ascertained the expression of TNFR-II after treatment with TNF-α in Ca9-22 cells. However, TNF-α did not induce TNFR-II expression in Ca9-22 cells. Therefore, we concluded that the effects of TNF-α are mediated through TNFR-I. TNF-α activates caspases and induces apoptosis in cells. However, C9-22 cells were alive during the experimental periods even after stimulation with TNF-α (Additional file 1: Figure S2). Therefore, we think that the apoptotic activity of TNF-α towards host cells does not affect *P. gingivalis* invasion.

ICAM-1 as well as Rab5 was associated with TNF-α-augmented *P. gingivalis* invasion (Figures 6 and 8). Adhesion of *P. gingivalis* to host cells is multimodal and involves the interaction of bacterial cell surface adhesins with receptors expressed on the surfaces of epithelial cells. Adhesion of *P. gingivalis* to host cells is mediated by many extracellular components, including fimbriae, proteases, hemagglutinins, and lipopolysaccharides (LPS). Among the large array of virulence factors produced by *P. gingivalis*, the major fimbriae (FimA), as well as cysteine proteinases (gingipains), contribute to the attachment to and invasion of oral epithelial cells [49,50]. On the other hand, integrins can act as receptors for the integrin-binding proteins of several bacterial species [51–53]. *P. gingivalis* also associates with β1 and α5β1 integrin heterodimers via FimA. αVβ3 integrin also mediates fimbriae adhesion to epithelial cells [48]. In addition, carbohydrate chains on epithelial cell membrane glycolipids have been reported to act as receptors for *P. gingivalis* [54]. It has been demonstrated that ICAM-1 is required for the invasion of *P. gingivalis* into human oral epithelial cells [36]. Various cytokines including TNF-α induce expression of ICAM-1 [55,56]. Therefore, ICAM-1 expresion and *P. gingivalis* invasion in periodontal sites may be associated with the primary stages of the development and progression of chronic periodontitis.

It has been demonstrated that a large number of intracellular bacteria are present in IL-6-treated cells that have an increasing amount of Rab5 [41]. These results indicate that overexpression of Rab5 by cytokines may promote the fusion of bacteria containing phagosomes with early endosomes and thereby inhibit their transport to lysosomes and may help in prolongation of bacterial survival in host cells and thus establish a chronic infection that could exacerbate the immune response. At periodontal sites, such phenomena could occur. Periodontopathic bacteria induce various cytokines including TNF-α. It has been shown that of TNF-α is upregulated in periodontitis, e.g., in gingival crevicular fluid [23] and in gingival tissues [24]. Therefore, periodontopathic bacteria including *P. gingivalis* induce the production of cytokines including TNF-α in periodontal tissues. Excess TNF-α in periodontal tissues activates gingival epithelial cells and increases the possibility of *P. gingivalis* invasion in the cells, resulting in persistence of *P. ginigvalis* infection and prolongation of immune responses in periodontal tissues.

## Conclusions

We demonstrated that *P. ginigvalis* invasion into human gingival epithelial cells was enhanced by stimulation with TNF-α. TNF-α in periodontal tissues, the production of which is induced by plaque bacteria including *P. gingivlis* and is increased by diabetes, may lead to persistent infection of *P. ginigvalis* and prolongation of immune responses in periodontal tissues.

## Methods

### Bacterial strains and growth conditions

*P. gingivalis* ATCC 33277 was used as a wild-type strain in this study. This strain was grown at 37°C under anaerobic conditions on 5% horse blood agar plates (Poa Media, Eiken Chemical Co., Ltd., Tokyo, Japan) and in 30 mg/ml trypticase soy broth (BD Biosciences, SanJose, CA) supplemented with 2.5 mg/ml yeast extract (BD Biosciences), 5 μg/ml hemin and 5 μg/ml menadione. Bacterial growth was monitored by measuring the optical density at 660 nm (OD660). For invasion assays, an inoculum with an infection ratio (multiplicity of infection [MOI]) of 100 bacteria per cell was added to the cell culture medium.

### Cell culture

The human gingival epithelial cell line Ca9-22 was obtained from RIKEN Bioresource Center (Ibaraki, Japan). Ca9-22 cells were cultured under standard conditions in Eagle's minimal essential medium (E-MEM; Wako Pure Chemical Industries, Ltd., Osaka, Japan) containing 10% fetal bovine serum (FBS), 1% penicillin and streptomycin at 37°C in a humidified atmosphere of 5% CO_2_. The monocytic cell line THP-1 was obtained from Japanese Collection of Research Bioresources Cell Bank (Osaka, Japan). THP-1 cells were cultured under standard conditions in Roswell Park Memorial Institute (RPMI) 1640 Medium (Invitrogen, Carlsbad, CA) containing 10% FBS, 1% penicillin and streptomycin at 37°C in a humidified atmosphere of 5% CO_2_.

### Antibodies

Antibodies were obtained from the following sources: antiserum for *P. gingivalis* whole cells was kindly donated by Dr. Fuminobu Yoshimura (Aichi-gakuin University, Aichi, Japan); mouse monoclonal antibody specific for ICAM-1, goat polyclonal antibody specific for ICAM-1, mouse monoclonal antibody specific for TNFRI, mouse monoclonal antibody specific for TNFRII and mouse immunoglobulin G (IgG) (R & D Systems, Minneapolis, MN); mouse monoclonal antibody specific for Rab5 (BD Biosciences); rabbit polyclonal antibody specific for ICAM-1 (Santa Cruz Biotechnology, Dallas, TX); goat IgG (Alpha Diagnostic Intl. Inc., San Antonio, TX); mouse monoclonal antibody specific for β-actin (Biovision Inc., Milpitas, CA); anti-rabbit IgG-Alexa 555 and anti-rabbit IgG-Alexa 633 (Invitrogen); mouse monoclonal antibody specific for GFP (Novus Biologicals, Littleton, CO), anti-mouse IgG-HRP, anti-rabbit IgG-HRP and mouse monoclonal antibody specific for β-actin (Cell Signaling Technology, Danvers, MA).

### Vector constructs

GFP-Rab5Q79L, GFP-Rab5WT, and GFP-Rab5S34N in pcDNA3 constructs were kindly provided by Dr. Yuji Yamamoto (Tokyo University of Agriculture, Tokyo, Japan) [57,58]. The GST-R5BD vector was kindly donated by Dr. Guangpu Li (University of Oklahoma Health Science Center, Oklahoma City, OK).

### *P. gingivalis* invasion assay

Invasion of bacteria was quantitated by a standard antibiotic protection assay as described previously [59]. Briefly, Ca9-22 cells were seeded in 12-well flat-bottom culture plates and were incubated overnight before administration of *P. gingivalis*. The cells then were washed twice with phosphate-buffered saline (PBS) and incubated for a further 1 h in OPTI-MEM (Invitrogen) without antibiotics. The cells were treated with 10 ng/ml of recombinant human TNF-α (Wako) for 3 h. *P. gingivalis* suspended in OPTI-MEM was added to the Ca9-22 cells at an MOI of 1:100 and further incubated at 37°C in 5% CO_2_ for 1 h. Unattached bacteria were removed by washing with PBS three times. OPTI-MEM containing 200 μg/ml of metronidazole and 300 μg/ml of gentamicin was added to the plates and they were incubated for 1 h. The cells were washed twice with PBS, and then 1 ml of sterile distilled water per well was added and the cells were suspended persistently by pipetting to disrupt them. The lysates were serially diluted and plated on 5% horse blood agar plates (Poa Media, Eiken Chemical) and then incubated anaerobically at 37°C for 10 days. Colony-forming units (CFU) of invasive *P. gingivalis* in cells were then enumerated.

### Silencing of Rab5 gene

Ca9-22 cells were transfected with 100 pmol siRNA specific for Rab5 (RAB5A-HSS108978, Invitrogen) or control siRNA (Stealth™ RNAi Negative Control Medium GC Duplex, Invitrogen) using Lipofectamine 2000 reagent, as described by the manufacturer (Invitrogen). Then, expression of Rab5 in the cells was examined by Western blotting using a monoclonal antibody to Rab5. Next, Rab5 siRNA-transfected Ca9-22 cells were incubated with *P. gingivalis* ATCC 33277 (MOI =100) for 1 h. Viable *P. gingivalis* in the cells was determined as described above.

### Immunostaining

Treated Ca9-22 cells were fixed with 4% formaldehyde for 10 min. Nonspecific binding of antibodies was blocked by incubation with 5% sheep serum in 10 mM Tris pH 7.6, 150 mM NaCl, and 0.05% Tween20 (TBS-T) for 1 h, and then the cells were incubated overnight at 4°C with a primary antibody (antiserum for *P. gingivalis* whole cells, mouse monoclonal antibody specific for ICAM-1) in TBS-T. After washing with buffer A (10 mM Tris pH 7.6, 300 mM NaCl, and 0.5% Tween20) 6 times, the cells were treated with a secondary antibody (anti-rabbit IgG-Alexa 555 or anti-mouse IgG-Alexa 555 and anti-rabbit IgG-Alexa 633) in buffer A for 1 h. Cells were then observed by a confocal laser scanning microscope (Leica microsystems, Welzlar, Germany). Some Ca9-22 cells were transfected with vectors containing genes of GFP alone (control), GFP-Rab5 (S34N) (inactive form of Rab5), and GFP-Rab5 (Q79L) (active form of Rab5). To clarify whether *P. gingivalis* cells are in the epithelial cells, a z-series with 0.5 μm-intervals was scanned and images of the x-z and y-z planes were reconstructed with the orthogonal section tool.

### Western blotting

TNF-α-treated and non-treated Ca9-22 cells and THP-1 cells were lysed in SDS-PAGE sample buffer, separated by SDS-PAGE, and transferred onto Immobilon-P Transfer Membranes (Millipore, Billerica, MA). The membranes were blocked with PVDF Blocking Reagent for Can Get Signal (Toyobo) in TBS-T for 1 h at room temperature and then incubated with antibodies to TNFRI, TNFRII, Rab5 and ICAM-1 overnight at 4°C. After washing 3 times with TBS-T, the membranes were incubated with horseradish-peroxidase-conjugated anti-rabbit or mouse IgG antibodies in Can Get Signal® Immunoreaction Enhancer Solution. The membranes were washed 3 times with TBS-T and then immunoreactive bands were visualized using ECL Western Blotting detection reagents (GE Healthcare, Uppsala, Sweden) or Immuno Star LD (Wako). The membranes were stripped and probed with anti-β-actin antibodies as a loading control.

### GST-R5BD pull-down assay

The GST-R5BD pull-down assay was based on the method described by Liu et al. [60]. Ca9-22 cells were transfected with GFP-Rab5 (WT) using Lipofectamine 2000 reagent, as described by the manufacturer (Invitrogen). The transfectants were pretreated with MAP kinase inhibitors, including a p38 inhibitor (SB203580, 5 μM), JNK inhibitor (SP600125, 1 μM), and ERK inhibitor (PD98059, 5 μM) (Calbiochem, San Diego, CA), or with an NF-κB inhibitor (PDTC, 5 μM) (Sigma-Aldrich, St. Louis, MO) at 37°C for 1 h followed by stimulating with 10 ng/ml TNF-α for 3 h. Thereafter, cell extracts were prepared in lysis buffer containing 25 mM HEPES pH 7.4, 100 mM NaCl, 5 mM MgCl_2_, 0.1% Nonidet P-40, 2% glycerol, 1 mM dithiothreitol, and protease inhibitors. The cell lysates were centrifuged at 13,000 × g for 10 min at 4°C, and then the supernatants were incubated with 20 μl of GST-R5BD bound to glutathione-Sepharose 4B beads for 10 min at 4°C under rotation. Thereafter, beads were collected and washed 3 times with lysis buffer. Samples were re-suspended in SDS sample buffer and analyzed by Western blotting.

### Measurement of cell viability

Cell viability was assessed by the trypan blue staining assay. Ca9-22 cells were preincubated with wortmannin (Wort, 300 nM) for 3 h or with actinomycin D (Act D, 1 μg/ml ), cycloheximide (CHX, 1 μg/ml ), NF-κB inhibitor (PDTC, 5 μM), MAP kinase inhibitors, including a p38 inhibitor (SB203580, 5 μM), JNK inhibitor (SP600125, 1 μM) and ERK inhibitor (PD98059, 5 μM), at 37°C for 1 h and were then incubated with TNF-α for 3 h. Viability of the cells was determined by an exclusion test with trypan blue. Each measurement was repeated three times independently. Those compounds were not toxic to the cells. (Additional file 2: Figure S1).

### Statistical analyses

All experiments were performed in triplicate for each condition and repeated at least three times. Statistical analyses were performed using an unpaired Student’s *t* test. Multiple comparisons were performed by one-way analysis of variance and the Bonferroni or Dunn method, with results presented as the mean ± standard deviation. P-values less than 0.05 were considered statistically significant.

## Additional files

Additional file 1: Figure S2. Numbers of alive *P. gingivalis* bacteria in Ca9-22 cell cultures. The numbers of intracellular and extracellular *P. gingivalis* were determined in Ca9-22 cells. Ca9-22 cells were treated with 10 ng/ml TNF-α for 3 h. The cells were infected with *P. gingivalis* (MOI 100) for 1 h. The cells were further cultured in media containing antibiotics for various time periods to kill extracellular bacteria. Then the cells were incubated in antibiotics-free media for 0–48 h, and the numbers of intracellular and extracellular bacteria were determined. The assays were carried out in triplicate as described in Methods. * and **, significantly different (*P* < 0.05 and *P* < 0.01, respectively) from the mean value for TNF (−). Error bars indicate standard errors of the means. Additional file 2: Figure S1. Cytotoxicity of chemical compounds used in this study. Ca9-22 cells were preincubated with wortmannin (Wort, 300 nM) for 3 h or with actinomycin D (Act D, 1 μg/ml ), cycloheximide (CHX, 1 μg/ml), an NF-κB inhibitor (PDTC, 5 μM) and MAP kinase inhibitors, including a p38 inhibitor (SB203580, 5 μM) (indicated as “SB”), JNK inhibitor (SP600125, 1 μM) (indicated as “SP”) and ERK inhibitor (PD98059, 5 μM) (indicated as “PD”), at 37°C for 1 h and were then incubated with TNF-α for 3 h. Viability of the cells was determined by an exclusion test with trypan blue.
