# Supplementary figures and images for: TNF-α augmented Porphyromonas gingivalis invasion in human gingival epithelial cells through Rab5 and ICAM-1
Source: BMC Microbiol. 2014 Sep 3;14:229. doi: 10.1186/s12866-014-0229-z (PMC4159534; doi:10.1186/s12866-014-0229-z)

## Slide 1
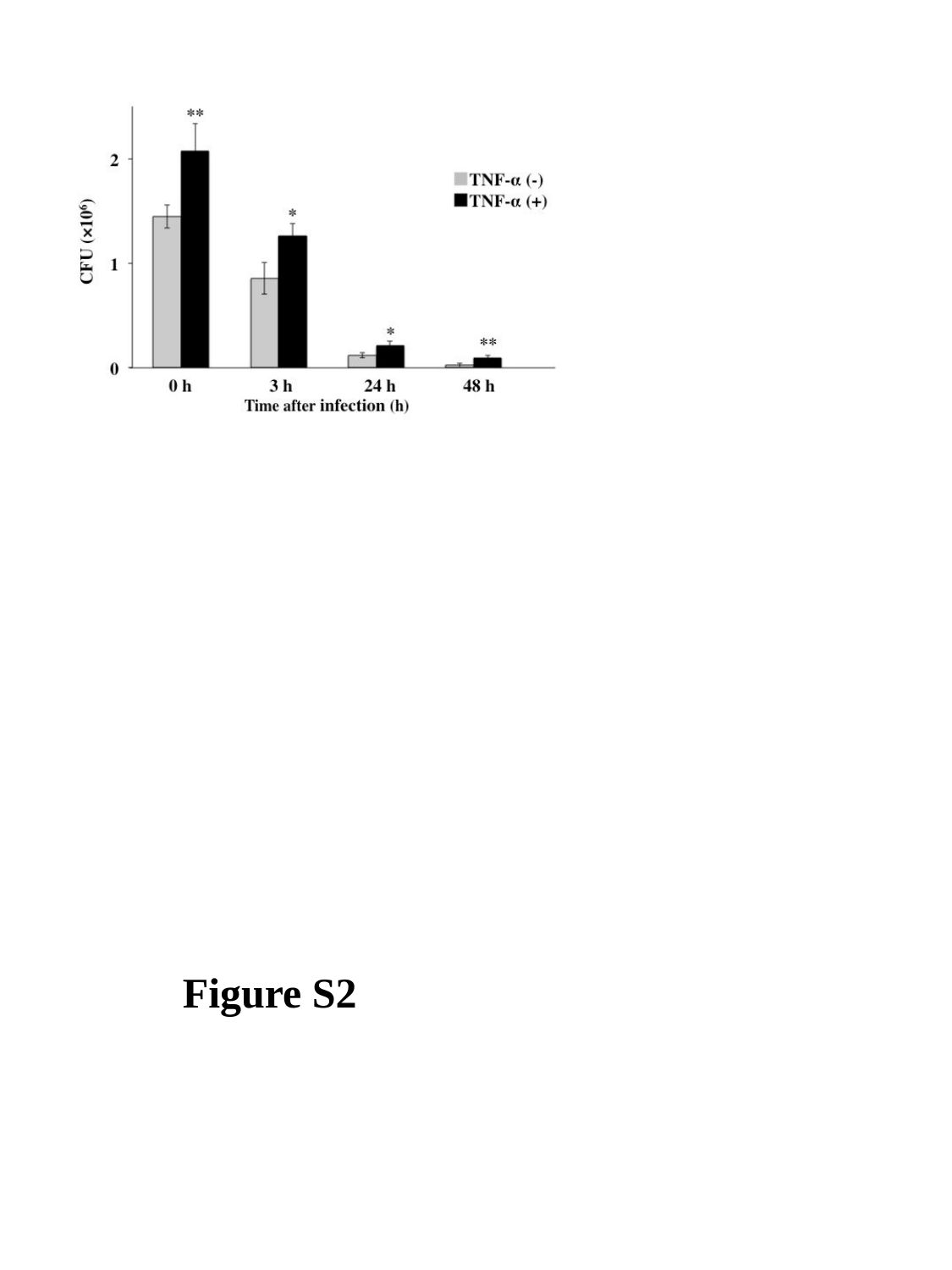

Supplement: Additional file 1: Figure S2. — Numbers of alive P. gingivalis bacteria in Ca9-22 cell cultures. The numbers of intracellular and extracellular P. gingivalis were determined in Ca9-22 cells. Ca9-22 cells were treated with 10 ng/ml TNF-α for 3 h. The cells were infected with P. gingivalis (MOI 100) for 1 h. The cells were further cultured in media containing antibiotics for various time periods to kill extracellular bacteria. Then the cells were incubated in antibiotics-free media for 0–48 h, and the numbers of intracellular and extracellular bacteria were determined. The assays were carried out in triplicate as described in Methods. * and **, significantly different (P < 0.05 and P < 0.01, respectively) from the mean value for TNF (−). Error bars indicate standard errors of the means. [file 12866_2014_229_MOESM1_ESM.pptx]

## Slide 1
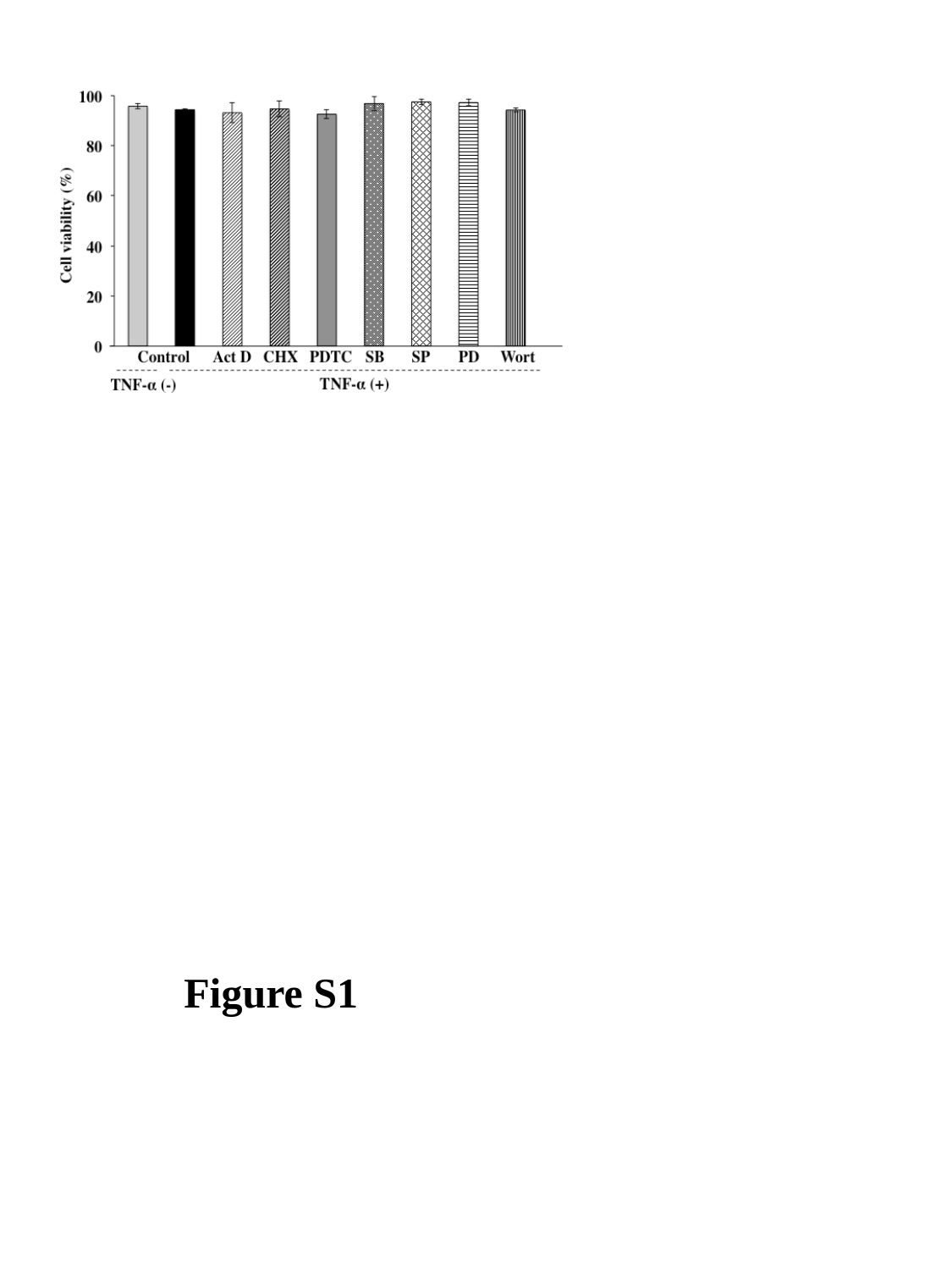

Supplement: Additional file 2: Figure S1. — Cytotoxicity of chemical compounds used in this study. Ca9-22 cells were preincubated with wortmannin (Wort, 300 nM) for 3 h or with actinomycin D (Act D, 1 μg/ml ), cycloheximide (CHX, 1 μg/ml), an NF-κB inhibitor (PDTC, 5 μM) and MAP kinase inhibitors, including a p38 inhibitor (SB203580, 5 μM) (indicated as “SB”), JNK inhibitor (SP600125, 1 μM) (indicated as “SP”) and ERK inhibitor (PD98059, 5 μM) (indicated as “PD”), at 37°C for 1 h and were then incubated with TNF-α for 3 h. Viability of the cells was determined by an exclusion test with trypan blue. [file 12866_2014_229_MOESM2_ESM.pptx]
